# The Safety of Long-term Daily Usage of a Proton Pump Inhibitor: A Literature Review

**DOI:** 10.7759/cureus.5563

**Published:** 2019-09-04

**Authors:** Mohammed K Abbas, Abdul Rehman Z Zaidi, Chris A Robert, Suyeewin Thiha, Bilal Haider Malik

**Affiliations:** 1 Internal Medicine, California Instititute of Behavioral Neurosciences and Psychology, Fairfield, USA; 2 Research, California Institute of Behavioral Neurosciences and Psychology, Fairfield, USA; 3 Obstetrics & Gynecology, California Institute of Behavioral Neurosciences and Psychology, Fairfield, USA; 4 Internal Medicine, California Institute of Behavioural Neurosciences and Psychology, Fairfield, USA; 5 Medicine, California Institute of Behavioral Neurosciences and Psychology, Fairfield, USA

**Keywords:** proton pump inhibitors, gastric cancer, stomach neoplasms, long term use, helicobacter pylori, omeprazole, pantoprazole

## Abstract

Proton pump inhibitors (PPIs) are amongst the most prescribed medications in the whole world due to their effectiveness and safety profile. However, doubts have arisen about its safety in long term use and have been associated with an increased risk of developing gastric cancer. We aim to study if there is an association between chronic PPI use and the risk of gastric cancer. If this is true, we would like to know the duration of use at which the risk of cancer is high. We performed a literature review of relevant full articles present in the PubMed database that were published in the last five years. Articles that were in the English language and discussed the risk of gastric cancer with chronic PPI use in adult age groups (18 years and above) were evaluated. Only observational or interventional studies with more than 20,000 participants were considered. Two nationwide based studies were included in this review, the Cheung study, and the Brusselaers study. The Cheung study included a total of 63,397 individuals, where 153 cases developed gastric cancer. PPI users had a hazard ratio of 2.44 (95% confidence interval [CI] 1.42-4.20), and the risk of cancer increased with the duration of PPI use. The Brusselaers study included a total of 797,067 individuals, where 2,219 cases developed gastric cancer. The standardized incidence ratio of gastric cancer among PPI users was 3.38 (95% CI 3.23-3.53), and the risk of cancer increased with the duration of PPI use. Therefore, chronic PPI use is associated with an increase in the risk of gastric cancer. It might also be an independent risk factor for gastric cancer.

## Introduction and background

Richard Dawkins once said: “It is possible in medicine, even when you intend to do good, to do harm instead. That is why science thrives on actively encouraging criticism rather than stifling it”. Today, proton pump inhibitors (PPIs) are among the most commonly prescribed medications [1]. For decades, PPIs succeeded in convincing the healthcare community worldwide regarding its effectiveness and its safety profile [2]. While the effectiveness part remains true, concerns have been raised regarding the safety of long term use of PPIs and the serious adverse effects it may cause. Increased risk for gastric cancer, which ranks third among cancer-related mortalities, has been suggested with long term use of PPIs [3].

**Figure 1:**
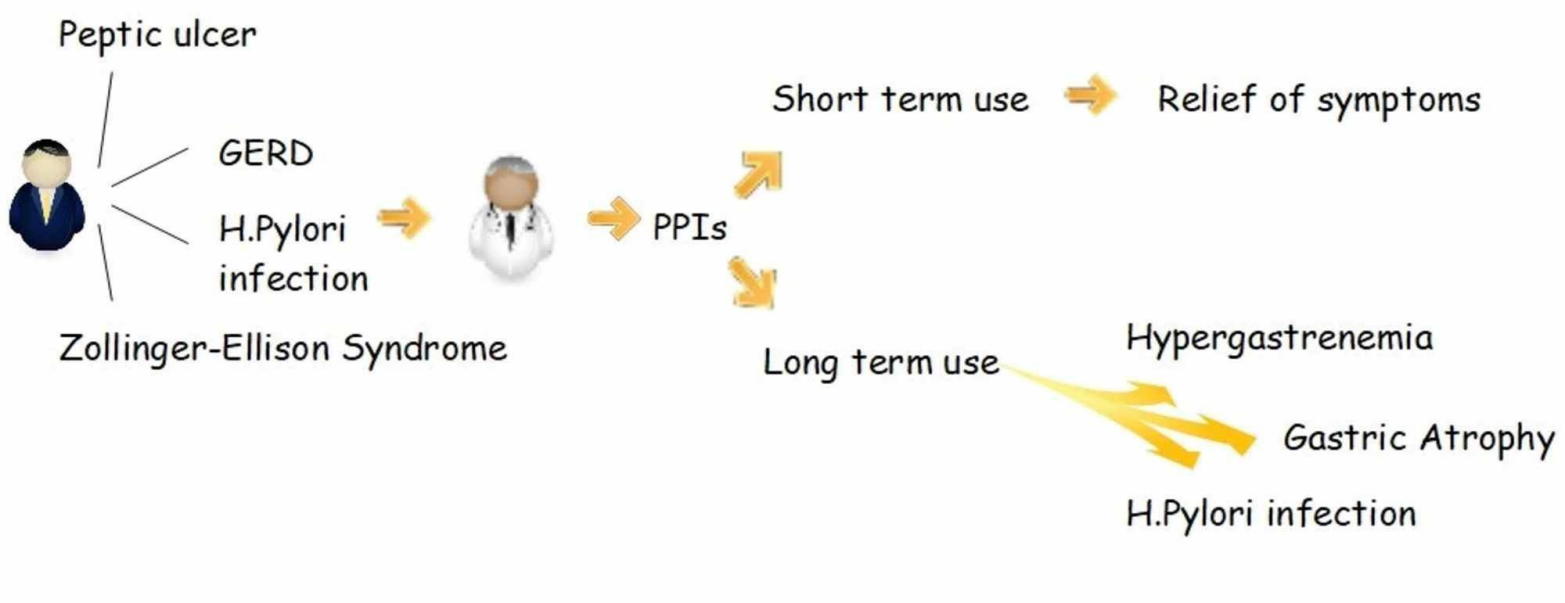
PPI and its effects of short-term and long-term use PPIs - proton pump inhibitors; GERD - gastroesophageal reflux disease, H. Pylori - Helicobacter pylori

PPIs were first made available in 1989, and till now they are the best in inhibiting gastric acid secretions and have been used mainly to treat gastroesophageal reflux disease, which affects up to 10% of the adult population daily [4]. Examples of other uses for PPIs are peptic ulcer disease, stress ulcer prophylaxis, Helicobacter pylori (H. pylori) eradication, stomach protection in nonsteroidal anti-inflammatory drugs (NSAIDs) chronic use, Zollinger-Ellison syndrome, upper gastrointestinal bleeding, esophagitis, and dyspepsia. Like any other drug, PPIs have known common minor adverse effects like headaches and gastrointestinal upset [5]. However recent studies have potentially linked long term use of PPIs to some systemic severe adverse effects like increased risk of osteoporosis-related fractures, Clostridium difficile infection, malabsorption of vitamins and minerals such as vitamin B12, calcium and iron, dementia, pneumonia, kidney disease, and stroke [6-8]. Besides that, some local effects of long term PPIs use include atrophic gastritis due to prolonged acid suppression, hypergastrinemia, chronic H. pylori infection, and development of gastric polyps [9, 10]. It is the local effects that led to concerns for increased risk of gastric cancer in patients on PPIs for an extended period. All four local effects are risk factors for gastric cancer. The dilemma we are facing is that although PPIs are used to treat patients with hypergastrinemia and H. pylori infection, in the long run, they cause hypergastrinemia and H. pylori infection (Figure 1).

Despite the above concerns, it is not all unfavorable for PPIs. Besides their pharmacologic superiority in decreasing gastric acid secretions compared to others, they also do that for an extended period. Therefore in most cases, they need to be taken only as a single daily dose. Some PPIs are cheap, readily available, and can be used over the counter. Although the last point may seem like an advantage, it leads to the critical problem of inappropriate use of the drug by ordinary people. Inappropriate use of PPI is also caused by physicians, who prescribe PPIs without any real indication for the patient, whether at an emergency or an office visit [11]. Add to that the inadequate, routine like recommendations of using PPIs by physicians in their discharge notes as well as the continuation of PPI therapy by primary care physicians without proper assessment leads to the unnecessary continuation of treatment, exposing individuals to the adverse effects and adding to their financial burden [12].

A few earlier studies researched the topic and suggested a possible link, but due to the small number of individuals tested and the presence of confounders, the results were not conclusive, and they recommended further studies to be done [13-15]. In this review, we will try to discover whether PPIs do cause gastric cancer. We will also try to define the long-term use at which there will be an increased risk of gastric cancer - is it one year, two years, three years, or more?

## Review

Methods

Specific steps were taken to ensure a quality review. Firstly we used PubMed as our single database of search. The search began on 5th May 2019. We wanted to identify studies that reported data on long term use of proton pump inhibitors and the risk of getting gastric cancer. We also searched for studies that included a definition of long-term use. The following keywords were used for our regular search: PPIs, gastric cancer, long term use, Helicobacter pylori, omeprazole, and pantoprazole. For our MeSH search, the keywords used were PPIs, stomach neoplasms, and Helicobacter pylori. Secondly, the following filters were applied to each of our searches: availability of full text, publications within the last five years, studies on humans only, articles in the English language, and age 19 years and above. These filters were used to help make sure that the articles used were up to date with current medical practice, could be properly analyzed and that the population included matched our specialty. The number of articles available per MeSH search using a single or combination of keywords can be found in Table 1.

**Table 1:** PubMed search results using MeSH keywords alone and in combination

MeSH keywords	Database	Number of articles
Proton Pump Inhibitors (PPIs)	PubMed	1,429
Helicobacter pylori	PubMed	1,633
Stomach neoplasms	PubMed	8,137
PPIs + Helicobacter pylori	PubMed	224
PPIs + stomach neoplasms	PubMed	67
Helicobacter pylori + stomach neoplasms	PubMed	413
PPIs + Helicobacter pylori + stomach neoplasms	PubMed	29

**Figure 2:**
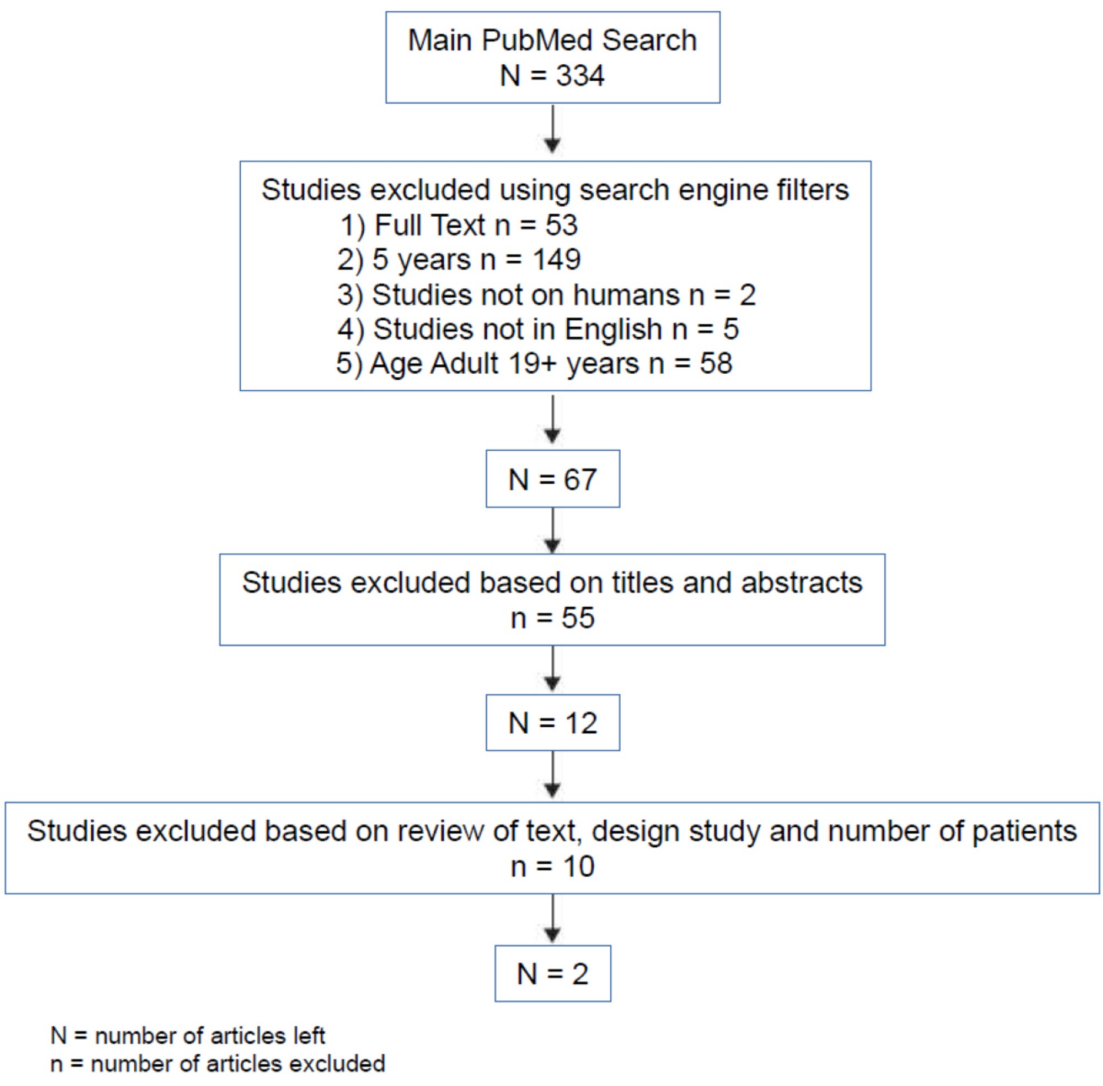
Flowchart of the process of articles selection

These articles were used along with their references for gathering relevant information about the topic. The main MeSH search of proton pump inhibitors and stomach neoplasms yielded 334 articles. After applying the above filters, the number came down to 67 articles. From there onwards, articles were reviewed by their titles and abstracts. Only 12 articles were closely related to our topic of search, and the remaining 55 articles were excluded. From the 12 articles, only observational and interventional studies with over 20,000 participants were included (Figure 2). A high number of participants was used to make sure the study power was sufficient, and conclusions reached resembles reality. All articles used were peer-reviewed, and no gray literature was used. All data were collected ethically.

The two studies included for analysis and comparison are both population-based cohort studies. The Cheung KS study published in 2017 was based on the population of Hong Kong, while the Brusselaers N study, also published in 2017 was based on the Swedish population [16, 17]. The purpose of the Cheung study was to see if there is an association between PPI use and gastric cancer in patients with H. pylori infection that have received triple therapy for H. pylori and successfully eradicated H. pylori. The duration of therapy was categorized into ≥1 year, ≥2 years and ≥3 years. Patients that developed cancer within one year of H. pylori eradication had been excluded. Regarding the Brusselaers study, the aim was to assess the risk of gastric cancer with PPI use with consideration to indications of use. The duration was set as the maintenance use of PPI for at least 180 days throughout the study period [18].

Results

Study Participants

Cheung study: A total of 63,397 individuals that fulfilled the inclusion criteria were included, 46.5% were men, and 53.5% were women. The median age at the time of successful triple therapy was 54.7 years, the median follow-up was 7.6 years, and the total follow-up duration was 483,260 person-years.

Brusselaers study: A total of 797,067 subjects that fulfilled the inclusion criteria were included, 41.5% were men, and 58.5% were women. The mean follow-up was 4.9 years, and the total follow-up duration was 3,866,836 person-years. Additional individuals that were taking histamine H2-receptor antagonists (H2RA), although mentioned in the study and were used for comparison, are not included in our study. Here we will focus on individuals taking PPIs only.

Risk of Gastric Cancer

Cheung study: A total of 153 individuals (0.24%) had developed gastric cancer even after H. pylori had been eradicated. PPI users (at least weekly use) had a higher risk of cancer (hazard ratio [HR] 2.44, 95% confidence interval [CI] 1.42-4.20) using propensity score adjustment with trimming. This result was supported with sensitivity analysis using both multivariable analysis (HR 2.19, 95% CI 1.31-3.66) and propensity score adjustment without trimming (HR 2.14, 95% CI 1.27-3.58), thus linking PPI use with the development of gastric cancer. After stratification by the site of the tumor, PPI use was found to increase risk of non-cardia cancer only (HR 2.59, 95% CI 1.42-4.72) and not the risk of cardia cancer (HR 1.97, 95% CI 0.57-6.82)

Brusselaers study: In this study 2,219 (0.28%) subjects developed gastric cancer during follow up. Standardized incidence ratios (SIRs) were 3.38 with 95% CI of 3.25-2.5 in all individuals exposed to PPIs. There was a similar increased risk for cardia (SIR 3.55, 95% CI 3.27-3.86) and non-cardia cancer (SIR 3.33, 95% 3.17-3.50). The risk was increased in all age groups, although young participants (≥19 age <40) had the highest increase (SIR 22.76, 95% CI 15.94-31.52). Because the study focused on the risk of gastric cancer per indication of the use of PPI, these results can be found in Table 2.

**Table 2:** Risk of gastric cancer in all individuals exposed to PPIs, stratified by indication PPIs - proton pump inhibitors; NSAID - nonsteroidal anti-inflammatory drug; SIR - standardized incidence ratio; CI - confidence interval Note: adapted from Brusselaers N, Wahlin K, Engstrand L, et al. [17]

Indications	Gastric cancer	
	Cases (n)/total	SIR (95% CI)
All	2,219 / 797,067	3.38 (3.25-3.53)
Gastro-oesophageal reflux	557 / 201,868	3.04 (2.80-3.31)
Peptic ulcers	721 / 79,597	8.75 (8.12-9.41)
Gastroduodenitis	350 / 104,955	3.68 (3.31-4.09)
Dyspepsia	136 / 43,901	3.07 (2.58-3.63)
Helicobacter pylori	440 / 58,366	9.76 (8.87-10.71)
No Helicobacter pylori	1,779 / 781,955	2.91 (2.78-3.05)
NSAID exposure	347 / 256,598	1.82 (1.64-2.03)
Only NSAID exposure	93 / 118,987	1.41 (1.14-1.73)
Aspirin exposure	872 / 293,590	2.42 (2.26-2.60)
Only aspirin exposure	252 / 126,863	1.93 (1.70-2.18)

Frequency and Duration of PPI Use and Risk of Gastric Cancer

Cheung study: A total of 3,271 (5.2%) individuals had used PPIs with a median duration of use of 2.7 years (interquartile range [IQR] 1.5-5.1 years). Regarding frequency, compared to the reference group (less than weekly use of PPIs) a progressive increase in the risk of gastric cancer was noticed with more frequent use of PPIs. For weekly use to less than daily use group, HR was 2.43 with 95% CI 1.37-4.31 whereas, for daily use group, HR was 4.55 with 95% CI 1.12-18.52. As for duration, ≥ 1 year of use group had an HR of 5.04 and 95% CI 1.23-20.61, ≥ 2 years of use group had an HR of 6.65 and 95% CI 1.62-27.26 and finally, ≥ 3 years of use group had an HR of 8.34 and 95% CI 2.02-34.41.

Brusselaers study: Here, long-term PPI users had a standardized incidence ratio (SIR) of more than threefold for gastric cancer (SIR of 3.38 with 95% CI 3.25-3.53). The risk was highest for those taking PPI < 1 year (SIR 12.82, 95% CI 12.19-13.47). The increased risk was present up to three years although to a lesser extent than those taking PPI < 1 year. At five years, the risk had decreased (Table 3).

**Table 3:** SIRs by estimated duration of use and 95% CIs of gastric cancer in those exposed to PPIs SIR - standardized incidence ratio, CI - confidence interval Note: reprinted from Brusselaers N, Wahlin K, Engstrand L, et al. [17]

Duration	Cases (n)/total	SIR (95%CI)
<1.0 year	1,552	12.82 (12.19-13.47)
1.0-2.9 years	2,193	2.19 (1.98-2.42)
3.0-4.9 years	1,098	1.10 (0.91-1.31)
≥5.0 years	153	0.61 (0.52-0.72)

Discussion

Mechanisms Linking PPIs and Gastric Cancer

PPIs are the most potent inhibitors of gastric acid secretion, and their inhibiting effect increases over time [19]. Several mechanisms can lead to the link between chronic use of PPIs and the development of gastric cancer. Past studies on rodents demonstrated that potent inhibition of gastric acid secretions had induced gastric cancer, associated with secondary hypergastrinemia that resulted in enterochromaffin-like cell hyperplasia (a carcinogenic effect) [20, 21]. This chain of events from blocking acid secretion to chronic hypochlorhydria to hypergastrinemia results in the proliferation of gastric mucosa, chronic inflammation, decrease in gastric mucosal glands, the emergence of intestinal glands and gastric atrophy [18]. Gastric atrophy, a precursor for gastric cancer, is usually associated with chronic H. pylori infection and there have been studies that confirm the fact that chronic PPIs use worsens gastric atrophy, thus increasing the risk for gastric cancer development [9, 22, 23]. However, these results may have been confounded to some extent by the lack of knowledge of the H. pylori status in the study population [13-15]. Prolonged use of PPI has also been linked to the formation of fundic polyps, another risk factor for gastric cancer [24]. All the changes mentioned above promote carcinogenesis, and in the presence of chronic hypochlorhydria, defense against pathogenic bacteria is weakened [25, 26]. This results in infections with bacteria such as Clostridium difficile and Campylobacter jejuni. These infections over time cause chronic inflammation and aid in the development of cancer. Furthermore, the increased gastric colonization of non-gastric bacteria produces nitrosamine - another carcinogenic substance to the gastric mucosa [27, 28].

Risk of Gastric Cancer in Individuals Using PPIs

Both studies provided evidence of increased risk of gastric cancer with prolonged PPI use despite different factors. Cheung study was able to establish the presence of the risk even in those individuals who were treated successfully from H. pylori infection (a strong risk factor for cancer on its own) as these individuals had a 2.4-fold increase compared to non-PPI users. The analysis also demonstrated a dose-dependent and time-dependent increase in the HRs of gastric cancer with PPIs use (HR 4.55 among daily PPI users and HR 8.34 for those taking PPI ≥3 years). On the other hand, the Brusselaers study provided evidence of increased risk while on PPIs maintenance dose regardless of the indication of use and presence or absence of local gastric risk factors (such as atrophy, hypergastrinemia and chronic H. pylori infection). The Brusselaers study showed a significant increase in gastric cancer in both cardia and non-cardia regions, whereas the Cheung study showed a significant increase only in the non-cardia region. The absence of a significant increase in the cardia region could be because of the relatively small number of cardia cancers (31 cases). The Brusselaers study showed an increased risk in all age groups studied; however, younger participants had the highest risk. This finding can be explained by the discovery of an increased prevalence of atrophic gastritis in Sweden due to the seroprevalence of H. pylori and obesity [29]. There have also been reports of higher risks of cancer in the young population, especially adenocarcinoma (diffuse type) [30, 31]. The Brusselaers study also showed that the risk for gastric cancer increased with the duration of use for the first three years, but at five years of use, the risk had dropped (SIR 0.61, 95% CI 0.52-0.72). This could be explained by the fact that PPI use may benefit patients who had gastrointestinal indications such as H. pylori, peptic ulcers, and hypergastrinemia. However, there is no explanation of why the risk decreased in individuals who had indications of PPI use that are not related to the gastrointestinal tract, such as protection from chronic aspirin and NSAIDs use.

Study Strengths

Cheung study: Firstly, a large number of participants were studied along with complete information about their diagnosis and drug prescription. This helps in increasing the study power and limits selection, information, and recall bias. Secondly, the presence of a long period of follow-up helps in better assessment of the long-term effect of PPIs. It is the first study to highlight that chronic PPI use, even after successful treatment of H. pylori, has been associated with a higher risk of gastric cancer. This has been achieved as only patients with successful treatment of H. pylori had been included. The study was also able to show a dose and time-response positive trend, hinting to the presence of a cause and effect relationship. Finally, the use of propensity score adjustment helped the control of potential confounders, and the use of various sensitivity analysis derived similar conclusions.

Brusselaers study: Again, here the vast number of study participants along with the availability of complete data on exposure, indications, and outcomes helps in increasing the study power. Add to that the long period of follow-up, which helps in better assessment of the effect of chronic PPI use on the individual. This study was also able to stratify the outcomes according to the different indications of PPI use which helped to give an idea of what the effects are of chronic PPI use in indications that have no increase in the risk of gastric cancer. Lastly, the consistency of results between sexes, different age groups, and all indication groups further strengthens the value of the results.

What is Next?

Although PPIs remain the best agent to control gastric acid secretion and are useful in many indications, especially those related to the gastrointestinal tract (such as peptic ulcers and H. pylori), their chronic use must be observed. The current practice of extensive use of PPIs, especially when there is no clear indication, should be revised and awareness among physicians regarding the possible complications associated with PPI use should grow to limit harm as much as possible to individuals in the future. The results from the above studies even challenge the chronic use of PPIs in individuals with proper indications of use. Due to the limitations of the above studies, we need more studies to be done keeping in mind the need to collect data for all the risk factors and to stratify the population tested to see the effects of different confounders. Future studies should also include individuals of different ages, ethnic groups, and socioeconomic statuses. Two of the findings mentioned in the Brusselaers study resulted in two new questions that need to be investigated. Firstly, why was there a higher risk of gastric cancer with chronic PPI use among the younger population, as compared to the older population? Secondly, why the risk of cancer dropped at five years of chronic use of PPIs, as compared to the increase in the risk of gastric cancer with chronic PPIs use during the first three years? We are also questioning the maximum duration of use of PPIs with the minimum risk of gastric cancer, and whether including few months of break from PPIs use in individuals that have been taking PPI for more than a year will help decrease the risk of gastric cancer. In the meantime, it is recommended to quickly investigate and treat individuals with H. pylori infection due to its profound effects. It is also recommended to raise awareness among physicians and patients of the importance to implement lifestyle modifications including weight loss and dietary modifications as this may help decrease the need for PPIs in some individuals.

Limitations

Both studies suffered from the inability to retrieve information about specific risk factors such as diet and family history from the electronic databases. Also, the effect of certain possible and important confounders such as smoking, alcohol intake, and obesity may have been underestimated as only severe consumption of alcohol, and morbidly obese patients may have been coded. Another problem is that data from both studies are limited to a specific ethnic group, and hence cannot be generalized for the rest of the world. This is because Chinese people are at a higher risk of developing gastric cancer compared to the western world. Mainly because of the increased prevalence of H. pylori infection as well as dietary patterns. Swedish people may be at a higher risk due to the increased prevalence of obesity, H. pylori infection, and familial history of gastric cancer [32].

## Conclusions

PPIs are the most potent agents in inhibiting gastric acid secretions. They are one of the most prescribed medications today and have many indications for use. However, chronic use has been shown to increase the risk of gastric cancer as presented by the Cheung and Brusselaers studies. This increase in risk is both dose and duration related. The increase in risk is also consistent among both sexes and in all age groups studied. Since PPIs are used in many individuals with no indication, it is recommended to limit PPI use to those with clear indications of use and to assess whether the indications still apply. It is also recommended to quickly treat individuals with local gastric risk factors for cancer such as chronic H. pylori infection and peptic ulcers. Besides, future studies are needed to fully explain the effect of chronic PPI use and the effect of present confounders. Future studies are also needed to define the maximum duration of use of PPIs, where the risk of gastric cancer is minimum and to answer the other questions mentioned above. In the end, we would like to thank the work done by all the researchers in this field and encourage others to continue pursuing the topic until we have all the answers.
